# M1 macrophage-targeted engineered ginseng stems and leaves-derived extracellular vesicles delivery system for alleviating rheumatoid arthritis

**DOI:** 10.1093/rb/rbag078

**Published:** 2026-04-26

**Authors:** Chuanjie Zhang, Yingjie Wang, Xiaoyu Jiang, Dake Wang, Yajiang Yuan, Jianye Li, Weiran Gao, Housen Jiang, Xifan Mei

**Affiliations:** Trauma and Emergency Center of the Third Affiliated Hospital of Jinzhou Medical University, Jinzhou 121000, China; Department of Ophthalmology, Beijing Chaoyang Hospital Affiliated to Capital Medical University, Beijing 100020, China; The Second Clinical Medical College of Shandong University of Traditional Chinese Medicine, Jinan 250014, China; Trauma and Emergency Center of the Third Affiliated Hospital of Jinzhou Medical University, Jinzhou 121000, China; Department of Medical Oncology, The First Affiliated Hospital of Jinzhou Medical University, Jinzhou 121001, China; Trauma and Emergency Center of the Third Affiliated Hospital of Jinzhou Medical University, Jinzhou 121000, China; Department of Medical Oncology, The First Affiliated Hospital of Jinzhou Medical University, Jinzhou 121001, China; Department of Hand Foot Orthopedics, First Affiliated Hospital of Shandong Second Medical University, Weifang 261000, China; Trauma and Emergency Center of the Third Affiliated Hospital of Jinzhou Medical University, Jinzhou 121000, China

**Keywords:** rheumatoid arthritis, plant-derived extracellular vesicles, targeted drug delivery, macrophage, CD44

## Abstract

Rheumatoid arthritis (RA) is a chronic autoimmune disease characterized by persistent synovial inflammation, oxidative stress damage and joint destruction. Current treatments often face challenges including limited targeting efficacy and systemic side effects. To develop a novel targeted therapy for RA, this study constructed a functionalized extracellular vesicle (EV) system by engineering ginseng stems and leaves-derived EVs with hyaluronic acid (HA) modification and curcumin (Cur) loading (Cur@EVs–PH). Structurally, the EVs–PH drug-loaded nanoplatform integrates the remarkable anti-inflammatory and antioxidant properties of EVs with the prolonged circulation capacity conferred by PEG. This design further capitalizes on the targeting ability of HA, thereby providing a robust structural foundation for the efficient delivery of therapeutics to disease sites. Our results demonstrated that the designed system achieved enhanced inflammatory targeting through CD44 receptor-mediated accumulation and exhibited potent anti-inflammatory and antioxidant activities. In the collagen-induced arthritis model, Cur@EVs–PH significantly alleviated joint swelling, reduced pathological scores and normalized immune organ indices. Mechanistic studies revealed that the therapeutic effects were mediated through suppression of pro-inflammatory cytokines and promotion of macrophage M2 polarization. This integrated strategy combining natural EVs, targeted modification and active drug loading provides a promising platform for the treatment of RA and other inflammatory diseases.

## Graphical Abstract

**Graphical Abstract:**
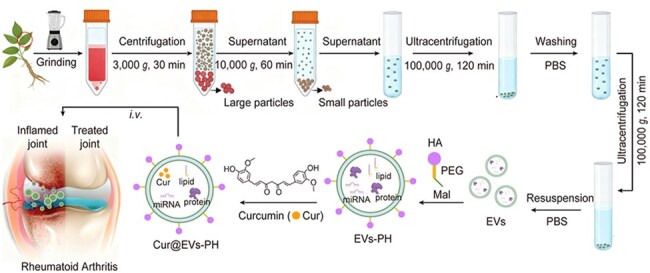

## Introduction

Rheumatoid arthritis (RA) is a chronic, systemic autoimmune disorder of unknown etiology and currently incurable [1, 2]. The disease is characterized by its high complexity, manifesting not only as joint pain, swelling and morning stiffness but also through potential multi-organ involvement [3, 4]. Significant heterogeneity in disease progression and treatment response among individuals makes clinical prediction challenging [5]. This imposes a multifaceted burden of suffering on patients, encompassing persistent chronic pain, progressive disability leading to functional loss and profound, often refractory fatigue [6, 7]. While contemporary treatment strategies, particularly the advent of biologic agents, represent a major therapeutic advance, they are accompanied by considerable limitations [8, 9]. Pharmacological management includes non-steroidal anti-inflammatory drugs (NSAIDs, e.g. ibuprofen), which provide rapid symptomatic relief but do not alter disease progression, and their long-term use carries risks of gastrointestinal complications and hepatorenal toxicity [10, 11]. Glucocorticoids (e.g. prednisone) offer potent anti-inflammatory effects; yet, chronic administration is associated with osteoporosis, metabolic disturbances, heightened infection risk and disease flare-ups upon withdrawal [12, 13]. Conventional synthetic disease-modifying antirheumatic drugs (csDMARDs, e.g. methotrexate) can slow structural joint damage; however, their slow onset of action, potential for hepatotoxicity and myelosuppression, and inadequate response or intolerance in a subset of patients pose significant constraints [14]. Biologic DMARDs [e.g. tumor necrosis factor (TNF)-α inhibitors], which target specific inflammatory pathways, demonstrate superior efficacy but are limited by high cost, parenteral administration and an increased susceptibility to serious infections (including tuberculosis reactivation) and immunogenic reactions [15, 16]. Consequently, the overall outlook for RA remains serious, underscoring a pressing need for the development of more precise, accessible and safer therapeutic strategies to improve patient outcomes and quality of life.

In recent years, plant-derived extracellular vesicles (P-EVs) have garnered significant attention as novel bioactive carriers [17, 18]. They exhibit inherent advantages such as high biocompatibility, low immunogenicity and the ability to efficiently cross biological barriers [19]. P-EVs can effectively encapsulate and deliver a diverse range of bioactive molecules—including proteins, nucleic acids and metabolites—demonstrating considerable potential in modulating immune responses, suppressing inflammation and promoting tissue repair [20, 21]. Notably, in the context of RA, a chronic immune-mediated inflammatory disorder, P-EVs offer promising therapeutic prospects by regulating aberrant immune cell functions and mitigating inflammatory joint damage [22]. Based on current research progress, EVs derived from ginseng present distinctive therapeutic advantages. Ginseng, a highly valued traditional herb, contains in its aerial parts—such as stems and leaves—a rich array of anti-inflammatory and immunomodulatory compounds, including ginsenosides, polysaccharides and trace elements [23, 24]. These bioactive constituents can be selectively encapsulated within EVs, enhancing their stability and targeted delivery [25]. Moreover, studies indicate that ginseng EVs can modulate macrophage polarization, inhibit the release of pro-inflammatory cytokines [e.g. TNF-α and interleukin (IL)-6] and restore Th17/Treg balance, thereby intervening in multiple key pathological pathways of RA [26, 27]. Furthermore, compared to ginseng roots, the stems and leaves represent a more renewable and abundant resource, which facilitates standardized and scalable production. This aspect addresses some of the limitations associated with conventional biologics, such as high manufacturing costs and complex purification processes [28]. Given their multifaceted immunoregulatory functions, natural carrier properties and sustainable sourcing, EVs emerge as a promising and translatable therapeutic strategy for RA.

CD44, as an important cell surface adhesion molecule, exhibits significantly high expression on the surface of activated macrophages [29, 30]. The upregulation of CD44 is primarily driven by abundant pro-inflammatory cytokines (such as TNF-α and IL-1β) within the local inflammatory milieu, positioning CD44 as a key receptor involved in inflammatory cell migration, adhesion and extracellular matrix remodeling [31, 32]. Consequently, it has emerged as a promising target for RA-specific therapies. Leveraging this targeting potential, hyaluronic acid (HA)—a naturally occurring linear polysaccharide—serves as a natural ligand for CD44 and can be used to functionalize nanoparticles [33, 34]. By conjugating or coating HA onto nanocarriers, the resulting system gains active targeting capability toward macrophages via specific HA–CD44 interactions. This enhances drug accumulation within inflamed joint tissues and improves therapeutic efficacy [35]. HA itself exhibits excellent biocompatibility, biodegradability, high hydrophilicity and lubricating properties, making it widely applicable in biomedical materials [36]. Beyond its role as a targeting moiety, HA is often employed as a stabilizer and functional component in drug carrier systems [37]. Moreover, it contributes intrinsic anti-inflammatory and immunomodulatory effects, thereby synergistically enhancing RA treatment [38]. Thus, a CD44-targeted nanodelivery system functionalized with HA not only achieves precise drug delivery but also exemplifies the multifunctional advantages of bioactive materials in RA therapy, offering a robust strategy for developing novel treatments with enhanced efficacy and reduced toxicity.

Curcumin (Cur), a natural polyphenolic compound derived from the rhizome of Curcuma longa, exhibits notable anti-inflammatory, antioxidant and immunomodulatory activities, showing considerable potential in the treatment of inflammatory diseases such as RA [39]. However, its poor aqueous solubility, rapid metabolism and low bioavailability significantly limit its clinical application [40]. In this study, Cur was selected as a model drug and encapsulated within EVs. To further enhance targeting efficiency, HA was covalently conjugated to the surface of the vesicles using polyethylene glycol (PEG) as a linker. PEG is non-toxic, non-immunogenic and non-antigenic and is known to prolong the circulation time of nanoparticles [41]. This strategy enabled the construction of a novel actively targeted delivery system. By leveraging both the innate drug delivery properties of plant-derived EVs and the specific binding capability of HA to CD44 receptors, this system achieves precise drug delivery to affected joints in RA.

## Materials and methods

### Extraction of ginseng stems and Leaves-Derived EVs

Fresh ginseng stems and leaves were washed with pre-chilled PBS, cut into small pieces and homogenized in PBS buffer containing protease inhibitors using a tissue homogenizer at a 1:3 (w/v) ratio on ice to thoroughly disrupt the tissue. The homogenate was then incubated in extraction buffer supplemented with 1% cellulase and 0.5% pectinase at 37°C for 1 h to degrade the cell wall. Subsequently, the mixture was subjected to differential centrifugation at 500 *g* for 10 min, 3000 *g* for 20 min and 10 000 *g* for 60 min to sequentially remove intact cells, large debris and organelles. The resulting supernatant was filtered through a 0.45-μm membrane and ultracentrifuged at 100 000 *g* for 120 min to pellet crude EVs. For further purification, the crude EV pellet was resuspended, layered on top of a discontinuous sucrose density gradient (60%, 45%, 30% and 15%), and centrifuged at 150 000 *g* for 2 h. The EVs enriched band at the 30%/45% interface was collected, diluted and washed by an additional ultracentrifugation step to remove sucrose [42].

The purified EV preparations were characterized by transmission electron microscopy (TEM) to confirm typical vesicular morphology, Nanoparticle tracking analysis (NTA) to determine particle size distribution, and Western blotting to verify the high expression of the positive EVs marker TET8 and the absence of negative markers (Actin, RbcL and Histone H3), thereby confirming their high purity and integrity.

### Surface modification of EVs with PEG-HA (PH)

The EVs were initially reacted with HA-PEG-maleimide (HA-PEG-Mal, Xi’an Rui Xi Biotechnology, China) at a molar ratio of 1:50 (EVs: HA-PEG-Mal) under room temperature in the dark for 2 h [43]. Unreacted PEG-Mal was subsequently removed using centrifugal filtration. The resulting pellet was resuspended in PBS to obtain the EVs–PH conjugate.

### Characterization of physicochemical properties of EVs and EVs–PH

#### Transmission electron microscopy

An appropriate amount of EVs or EVs–PH sample was applied to a copper grid, negatively stained with 2% phosphotungstic acid and air-dried at room temperature. The morphology and size of the particles were then observed under a TEM (JEM1200EX; JEOL, Tokyo, Japan).

#### Nanoparticle tracking analysis (Particle Metrix ZetaView)

Samples of EVs and EVs–PH were appropriately diluted with PBS and analyzed using a nanoparticle tracking analyzer to determine their particle size distribution and average diameter.

#### Stability assessment

For stability evaluation, samples were stored at 4°C and randomly divided into eight aliquots. One aliquot was randomly selected each day for NTA analysis to monitor changes in particle size and concentration. For lyophilization-reconstitution stability test, samples were mixed with 5% trehalose, pre-frozen and subsequently lyophilized. For reconstitution, PBS of the original volume was added, followed by vortex mixing. Post-reconstitution changes in particle size, morphology and aggregation were evaluated using NTA and TEM.

### The preparation of Cur@EVs–PH

Cur was dissolved in a 1:1 mixed solution of ethanol and acetonitrile, and then mix it with EVs–PH in PBS at a ratio of 10:1 by mass to EVs protein. Cur@EVs–PH was prepared using an intermittent ultrasonic bath (40 kHz, 100 W) for 15 minutes, followed by purification via three rounds of centrifugation at 5000 rpm using a 100-kDa ultrafiltration tube [44].

Encapsulation efficiency, EE (%) = (total initial curcumin − free unloaded curcumin)/total initial curcumin × 100%

Drug loading, DL (%) = (weight of encapsulated curcumin/total weight of curcumin-loaded EVs) × 100%

### Isolation and treatment of macrophages

First, 2 mL of 6% thioglycolate medium was intraperitoneally injected into mice for pre-stimulation over 3 days. The mice were then euthanized, and under aseptic conditions, 10 mL of pre-cooled PBS was injected into the peritoneal cavity. After a gentle abdominal massage, the peritoneal lavage fluid was aspirated. The collected lavage fluid was centrifuged at 1100 rpm for 5 minutes, and the supernatant was discarded. The pellet was resuspended in red blood cell lysis buffer to lyse erythrocytes, followed by termination of lysis with PBS and another round of centrifugation. The cells were resuspended in complete medium, counted and seeded into culture plates. They were incubated at 37°C with 5% CO_2_ for 4 h, after which non-adherent cells were gently removed by washing with PBS. After 24 h of culture, the cells were treated with 100 ng/mL Lipopolysaccharide (LPS) for an additional 24 h to induce polarization toward the M1 phenotype. The cells were then divided into the following treatment groups: PBS, EVs (100 μg/mL, 12 h), EVs–PH (100 μg/mL, 12 h) and Cur@EVs–PH (100 μg/mL, 12 h).

### Animal experimentation

SPF-grade DBA/1 mice (8-week-old) were housed in individually ventilated cages under strictly controlled environmental conditions, including a temperature of 22 ± 2°C, relative humidity of 55 ± 10% and a 12-/12-h light/dark cycle. Autoclaved bedding, food and water were provided *ad libitum*. Health status was monitored regularly in accordance with institutional guidelines for SPF animal maintenance. All animal experiments in this section have passed the ethical review of animal welfare at Jinzhou Medical University and comply with relevant national regulations on animal welfare ethics. The ethics number is JZMU-20250044.

### Collagen-induced arthritis mouse model

Using 8-week-old healthy DBA/1 mice, the dorsal tail base area was shaved prior to immunization. On day 0 of modeling, an emulsion of chicken-derived type II collagen thoroughly emulsified on ice was mixed with an equal volume of complete Freund’s adjuvant. A total of 100 µL of the emulsion, containing 100 µg of collagen per mouse, was administered via subcutaneous multipoint injection intradermally at the tail base and along both sides of the dorsal spine for primary immunization. On day 21, a booster immunization was performed using the same dose of type II collagen emulsified with incomplete Freund’s adjuvant, injected in the same manner. Mice were subsequently monitored for arthritic symptoms such as erythema and swelling in the paws, and clinical scores were recorded regularly throughout the observation period.

Collagen-induced arthritis (CIA) mice were randomly divided into four groups and administered the following treatments via intravenous injection (100 µL per dose, all formulations containing 10^12^ particles/mL): PBS, EVs, EVs–PH and Cur@EVs–PH.

### Assessment of CIA mice

After the 28th day of the first immunization, the swelling degree of the hind limbs of the mice was measured every 2 days, and the weight changes were recorded. The severity of arthritis lesions in mice was scored based on the degree of redness, swelling and deformity of the affected joints, and the arthritis score was the sum of the scores of the four joint lesions, with a maximum score of 16 points. The specific criteria are as follows: 0 points, no obvious redness, swelling or deformity; individuals with mild redness and swelling in a single toe joint will receive 1 point; if there is redness and swelling in the toe joint and plantar area, 2 points will be awarded; individuals with redness and swelling of the toe joints and feet accompanied by slight deformities will receive 3 points; those who experience severe redness, swelling and deformities in the toe joint, foot claw and ankle joint will receive 4 points. After euthanizing the mice, the spleen and thymus were weighed, and the spleen index and thymus index were calculated separately. Two independent investigators performed daily arthritis scoring, blinded to treatment allocation.

### Tissue sectioning and pathological evaluation

Tissue sections were prepared from inflamed joint specimens embedded in paraffin and sliced into 5 μm serial sections. Histological staining was carried out using hematoxylin–eosin (H&E) and Safranin O–Fast Green (SO/FG) to evaluate joint pathology under light microscopy. Pathological changes were scored based on four criteria: inflammatory cell infiltration, synovial hyperplasia, cartilage and bone damage, and angiogenesis (refer to Supplementary Table S1). All assessments were conducted by blinded pathologists using anonymized samples.

### Quantitative real-time polymerase chain reaction

Total RNA was extracted using TRIzol reagent, and its concentration was measured with a NanoDrop spectrophotometer. Subsequently, 1 µg of RNA was reverse-transcribed into cDNA using a reverse transcription kit under the following conditions: 42°C for 15 minutes and 85°C for 5 minutes. A 20-µL quantitative polymerase chain reaction (qPCR) reaction mixture was prepared, containing 10 µL of 2× SYBR Green Master Mix, 0.4 µL each of forward and reverse primers (10 µM), 2 µL of cDNA and ddH_2_O to adjust the final volume. Relative gene expression was calculated using the ΔΔCt method, with GAPDH serving as the internal reference for statistical analysis. The sequences of the primers used are listed in Supplementary Table S2.

### Western blot

Protein samples were lysed and extracted using RIPA lysis buffer (containing 1% protease inhibitor), and the supernatant was collected. Protein concentration was determined using the BCA assay, followed by denaturation. Proteins were separated by SDS-PAGE gel electrophoresis and subsequently transferred onto a PVDF membrane. The membrane was blocked with 5% skim milk, followed by incubation with a primary antibody (CD44, Proteintech, China) at 4°C overnight and an HRP-conjugated secondary antibody at room temperature for 1 hour. After antibody incubation, the membrane was washed three times with TBST for 5 minutes each. Finally, signals were detected using a chemiluminescent substrate, and images were captured with an imaging system and analyzed using ImageJ software.

### Immunofluorescence

Cells from different treatment groups or frozen tissue sections were fixed with 4% paraformaldehyde for 10 minutes, followed by three washes with PBS (5 minutes each). Permeabilization was performed using 0.3% Triton X-100 for 10 minutes. Blocking was carried out with 5% normal goat serum (or serum from the corresponding species) at room temperature for 30 minutes. Primary antibodies (CD44, CD86, CD206) were applied and incubated overnight at 4°C in a humidified chamber. The next day, samples were washed three times with PBS (5 minutes each). Fluorescently labeled secondary antibodies were added and incubated for 1 hour in the dark, followed by another three washes with PBS (5 minutes each). Nuclei were stained with DAPI (1 µg/mL) for 5 minutes and washed afterward. Slides were mounted using anti-fade mounting medium (containing anti-fading agents) and observed under a confocal microscope for image acquisition.

### Enzyme-linked immunosorbent assay

The concentrations of IL-1β, IL-6, TNF-α, IL-10 and IL-4 were quantified using commercial enzyme-linked immunosorbent assay kits (Solarbio, Beijing, China) according to the manufacturer’s instructions. Briefly, standards, samples, specific antibodies and HRP-conjugated streptavidin were sequentially added to the pre-coated wells and incubated at 37°C. After thorough washing to remove unbound components, a chromogenic substrate was added and allowed to develop at 37°C in the dark. The reaction was terminated using a stop solution, and the optical density was immediately measured at 450 nm using a microplate reader. Protein concentrations were determined by extrapolation from the standard curve.

### Flow cytometry

For uptake rate detection: EVs and EVs–PH were labeled with PKH26 (Beyotime, China), then co-incubated with M1-type macrophages for 4 h. After incubation, the macrophages were washed three times with PBS. The cells were then detached to form a single-cell suspension. Fluorescence signals were acquired using a flow cytometer, and the fluorescence intensity was analyzed using FlowJo software. All procedures were performed under light-protected conditions.

For macrophage polarization analysis: cells from different treatment groups were prepared as a single-cell suspension, counted and adjusted to a concentration of 1 × 10^6^ cells/mL. Fluorescently labeled monoclonal antibodies (CD206, BioLegend, USA) were added, and the cells were incubated for 30 minutes in the dark. Staining was terminated, and the cells were resuspended in 300 µL of flow cytometry buffer. 7-AAD was added to exclude dead cells. Fluorescence signals were acquired using a flow cytometer and analyzed with FlowJo software.

### Statistical analysis

All results were presented as mean ± SEM, and *P*-values < 0.05 were considered statistically significant. Statistical analyses were conducted using GraphPad Prism 9. Specifically, we first evaluated the normality of data distributions within each group using the Shapiro–Wilk test. When data met the normality assumption, we applied the independent samples Student’s *t*-test; however, for non-normally distributed data, we utilized the non-parametric Mann–Whitney *U* test for group comparisons.

## Results

### Characterization of physicochemical properties of EVs

**Figure 1:**
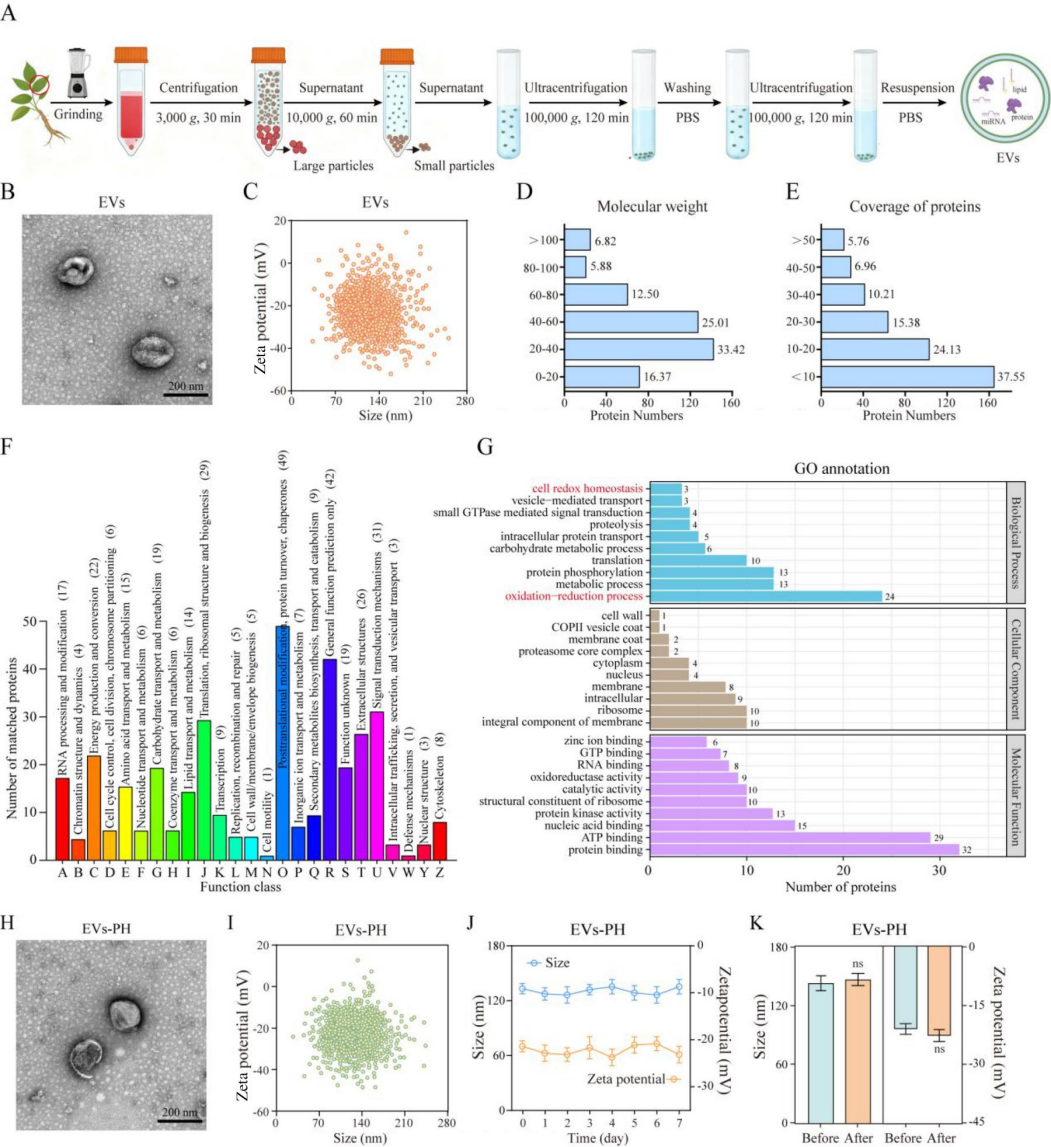
Characterization of physicochemical properties of EVs. (**A**) Schematic diagram of EVs extraction. (**B**) TEM micrograph showing the morphology of EVs. (**C**) Particle size and surface charge characterization of EVs. (**D**) The molecular weight distribution of EV proteins. (**E**) Protein sequence coverage distribution in EVs. (**F**) KEGG enrichment analysis of the EV proteome. (**G**) GO enrichment analysis of the EV proteome. (**H**) TEM micrograph showing the morphology of EVs–PH. (**I**) Particle size and surface charge characterization of EVs–PH. (**J**) Stability assessment of EVs–PH over a 7-day period (*n* = 3). (**K**) Stability assessment of EVs–PH during the lyophilization-reconstitution process (*n* = 3). Data are mean ± SEM. Statistical analysis was done using two-tailed unpaired *t*-tests. ns: not significant.

To explore the development potential of EVs derived from ginseng stems and leaves (EVs), we systematically characterized their physicochemical properties, protein composition and functional activities. EVs were first isolated by differential centrifugation (Figure 1A). The purified EVs were subsequently subjected to purity validation. Under equal protein loading conditions (20 μg), the cytosolic marker Actin, chloroplast marker RbcL and nuclear marker Histone H3 were readily detectable in the control total lysate, whereas the EVs-positive marker TET8 exhibited relatively weak expression. In contrast, the purified EV samples displayed a markedly different pattern (Supplementary Figure S1A and B). These results demonstrate that our isolation protocol effectively removes organelle contaminants, yielding highly purified EV preparations. Furthermore, surface functionalization was employed to enhance their targeting capability and stability. Morphological and physicochemical analyses revealed that the EVs exhibit a typical cup-shaped vesicular morphology under transmission electron microscopy (Figure 1B). Subsequent NTA confirmed that the hydrodynamic size was consistent with the TEM observations, with a homogeneous particle size distribution and an average diameter of 124.6 nm. And the Zeta potential was measured at −21.7 mV, indicating good colloidal stability (Figure 1C). Proteomic analysis identified more than 450 proteins within the EVs. Over 50% of these proteins had molecular weights between 20 and 60 kDa (Figure 1D), and the sequence coverage was generally below 10% (Figure 1E). Functional enrichment analysis indicated that these proteins are involved in key biological processes such as intracellular protein transport, carbohydrate metabolism and protein phosphorylation (Figure 1F). Notably, a substantial number of proteins were associated with the regulation of redox homeostasis, suggesting strong antioxidant potential of the EVs (Figure 1G). To enhance targeting capability and leverage this antioxidant activity, we conjugated HA to the surface of the EVs using a PEG-based coupling strategy, constructing an EVs–PH complex. The modified EVs–PH maintained intact vesicular morphology (Figure 1H) with an average size of approximately 127.3 nm and a Zeta potential of −23.4 mV (Figure 1I). No significant alterations in physicochemical properties were observed compared to unmodified EVs, indicating that the functionalization process did not compromise structural integrity. Stability assessments further demonstrated that EVs–PH retained their original characteristics after 7 days of storage in PBS at 4°C (Figure 1J) and maintained structural and functional integrity following lyophilization and reconstitution (Figure 1K). In summary, EVs–PH not only preserve the inherent antioxidant capacity of the original EVs but also acquire inflammation-targeting ability through HA modification, along with excellent stability. These features highlight the promising clinical translation potential of EVs–PH in antioxidant therapies and targeted delivery applications for inflammatory conditions.

### EVs–PH actively targets M1-type macrophages and enhances their phagocytic capacity

**Figure 2:**
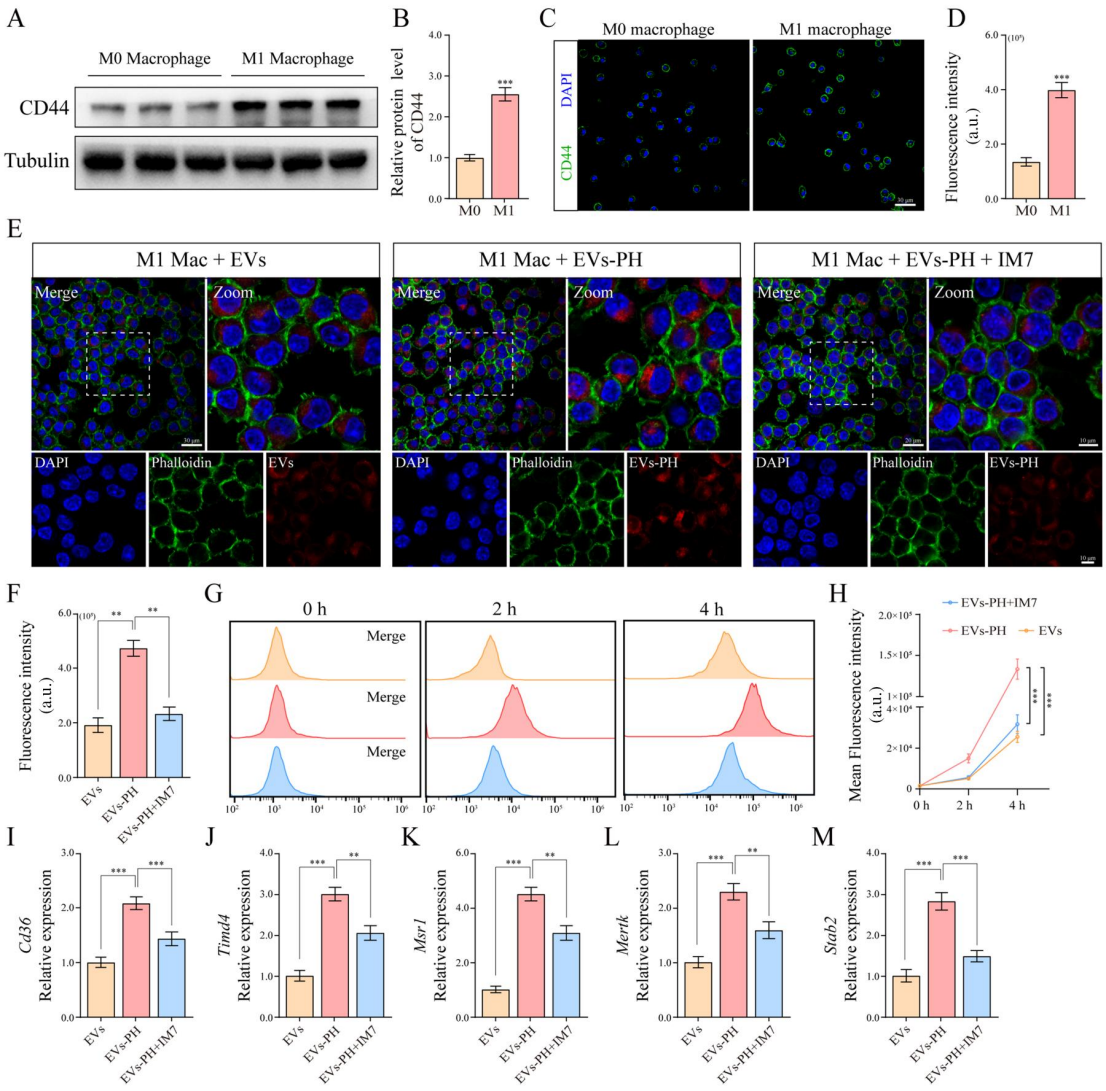
EVs–PH actively targets M1-type macrophages and enhances their phagocytic capacity. (**A**) Differential expression of CD44 between M0 and M1 macrophages as determined Western blot. (**B**) Quantitative Western blot analysis of CD44 in Figure 2A (*n* = 3). (**C**) Differential expression of CD44 between M0 and M1 macrophages was assessed by immunofluorescence. (**D**) Quantitative analysis of CD44 fluorescence intensity in Figure 2C (*n* = 6). (**E**) Immunofluorescence analysis of EVs and EVs–PH uptake by M1 macrophages. (**F**) Quantitative analysis of EVs and EVs–PH fluorescence intensity in Figure 2C (*n* = 6). (**G**) The dynamic uptake of EVs and EVs–PH by M1 macrophages was analyzed using flow cytometry. (**H**) Quantitative statistical analysis of Figure 2G (*n* = 6). (**I–M**) expression changes of phagocytic receptors (*Cd36*, *Timd4*, *Msr1*, *Mertk* and *Stab2*) in M1 macrophages among various groups (*n* = 6). Data are mean ± SEM. Statistical analysis was done using two-tailed unpaired *t*-tests. **P < 0.01, ***P < 0.001.

Targeting capability is a crucial indicator for evaluating nanocarriers. To validate the superior inflammatory targeting ability of EVs–PH, we first examined the expression of CD44—an HA receptor highly expressed on M1 macrophages, which play a key pro-inflammatory role in various diseases. Western blot (Figure 2A and B) and immunofluorescence (Figure 2C and D) results confirmed high CD44 expression on M1 macrophages, providing the basis for EVs–PH targeting. Subsequent visualization and quantitative comparison revealed significantly enhanced uptake of EVs–PH over unmodified EVs in inflammatory macrophages (Figure 2E and F). Flow cytometric monitoring further demonstrated that this uptake occurred rapidly at early stages (Figure 2G and H). Importantly, when M1 macrophages were pre-treated with the CD44 inhibitor IM7, the cellular internalization of EVs–PH was markedly suppressed, confirming that the enhanced and rapid uptake is primarily mediated through CD44 receptor recognition (Figure 2E–H). Previous studies suggest that HA binding to CD44 can substantially promote cellular internalization, which may explain the accelerated uptake of EVs–PH. To explore the underlying mechanism, we investigated the expression of efferocytosis-related and phagocytic receptors (Figure 2I–M) in M1 macrophages following EVs–PH treatment. The results demonstrated that EVs–PH significantly upregulate the expression of these receptors. In summary, these findings indicate that EVs–PH exhibits excellent cellular uptake efficiency, underpinning its active targeting capability and functional efficacy, which supports its potential for targeted therapeutic applications.

### EVs–PH alleviates oxidative stress and inflammation in M1 macrophages

**Figure 3:**
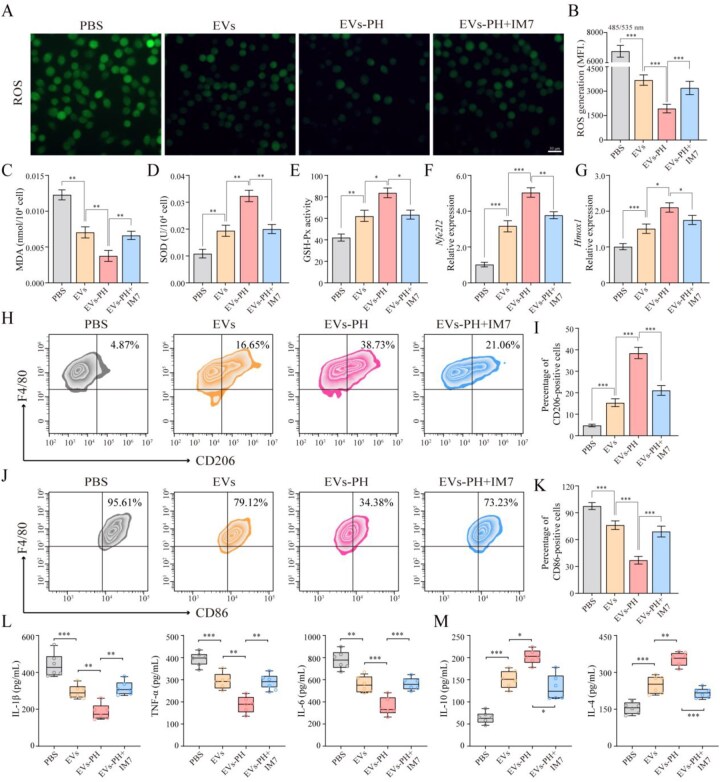
EVs–PH Alleviates oxidative stress and inflammation in M1 macrophages. (**A**) Immunofluorescence analysis of ROS in M1 macrophages across various groups. (**B**) Quantitative analysis of ROS fluorescence intensity in Figure 3A (*n* = 6). (**C**) Quantification of MDA in M1 macrophages across various groups (*n* = 6). (**D**) Quantification of SOD in M1 macrophages across various groups (*n* = 6). (**E**) Quantification of GSH-Px in M1 macrophages across various groups (*n* = 6). (**F**) Differential expression of *Nfe2l2* in M1 macrophages among different groups (*n* = 6). (**G**) Differential expression of *Hmox1* in M1 macrophages among different groups (*n* = 6). (**H**) The proportion of M2 macrophages in each group was determined by flow cytometry. (**I**) Quantitative statistical analysis of Figure 3H (*n* = 6). (**J**) The proportion of M1 macrophages in each group was determined by flow cytometry. (**K**) Quantitative statistical analysis of Figure 3J (*n* = 6). (**L**) Comparative analysis of pro-inflammatory factors (IL-1β, TNF-α and IL-6) expression in M1 macrophages from various groups (*n* = 6). (**M**) Comparative analysis of anti-inflammatory factors (IL-4 and IL-10) expression in M1 macrophages from various groups (*n* = 6). Data are mean ± SEM. Statistical analysis was done using two-tailed unpaired *t*-tests. *P < 0.05, **P < 0.01, ***P < 0.001.

To evaluate the regulatory effects of EVs–PH on oxidative stress and inflammatory status in M1-type macrophages, we induced M1 polarization in primary macrophages using LPS and compared four experimental groups: PBS, EVs, EVs–PH and EVs–PH + IM7. The results showed that, compared with the PBS group, EV treatment moderately reduced the levels of reactive oxygen species (ROS) (Figure 3A and B) and malondialdehyde (MDA) (Figure 3C) in M1 macrophages, indicating a certain antioxidant capacity. Notably, the EVs–PH group exhibited more pronounced improvements in these metrics. Antioxidant enzymes such as superoxide dismutase (SOD) and glutathione peroxidase (GSH-Px) were elevated following EVs treatment, with EVs–PH inducing a more significant upregulation (Figure 3D and E). These findings demonstrate the superior antioxidant efficacy of actively targeted EVs–PH. We further observed that both EVs and EVs–PH alleviated the LPS-induced suppression of the core antioxidant transcription factor *Nfe2l2* (encoding Nrf2) and its downstream target heme oxygenase-1 (*Hmox1*) (Figure 3F and G). These results suggest that the antioxidant effect of EVs–PH may be mediated through activation of the Nrf2/HO-1 pathway. Flow cytometry analysis revealed that LPS stimulation induced a typical M1 polarization state in macrophages, as evidenced by over 95% of cells being CD86-positive and <5% CD206-positive. Treatment with EVs partially restored the polarization balance, reducing the proportion of CD86-positive cells to below 79% and increasing CD206-positive cells to over 15%. More importantly, EVs–PH treatment more effectively suppressed M1 polarization while promoting M2 polarization, with CD86-positive cells decreasing to <35% and CD206-positive cells increasing to over 38%. However, when CD44 was blocked by IM7, the antioxidant, anti-inflammatory and M2-polarizing effects of EVs–PH were substantially attenuated, confirming that its functional benefits depend on CD44-mediated targeting and signaling (Figure 3H–K). In terms of inflammatory regulation, EVs–PH significantly downregulated the expression of pro-inflammatory cytokines (IL-1β, TNF-α and IL-6) (Figure 3L) and enhanced the secretion of anti-inflammatory factors (IL-4 and IL-10) (Figure 3M). In conclusion, EVs–PH enhances the regulation of M1 macrophages through CD44-targeted mechanisms, effectively activating intracellular antioxidant pathways, alleviating inflammation and facilitating phenotypic switching. These results underscore the therapeutic potential of EVs–PH, with CD44 playing a critical role in its mode of action.

To further verify whether EVs–PH exhibits dose‑dependent effects, we treated LPS‑induced macrophages with five concentrations of EVs–PH (0, 20, 50, 100 and 200 μg/mL, based on EVs protein content). Measurements of oxidative stress markers (ROS, MDA, SOD, GSH-Px) (Supplementary Figure S2A–E), inflammatory cytokine secretion (IL‑1β, TNF‑α, IL‑6, IL‑4, IL‑10) (Supplementary Figure S2F and G) and macrophage polarization markers (iNOS, Arg‑1) (Supplementary Figure S2H and I) revealed a clear dose–response relationship. From 0 to 100 μg/mL, increasing concentrations progressively alleviated oxidative stress, suppressed pro‑inflammatory cytokines and promoted a shift from M1 to M2 polarization. However, at 200 μg/mL, the therapeutic effects did not differ significantly from those at 100 μg/mL, indicating that the response had reached a plateau, consistent with a saturating dose (Supplementary Figure S2A–I).

### Active targeting capability of EVs–PH toward inflammatory sites in CIA mice

**Figure 4:**
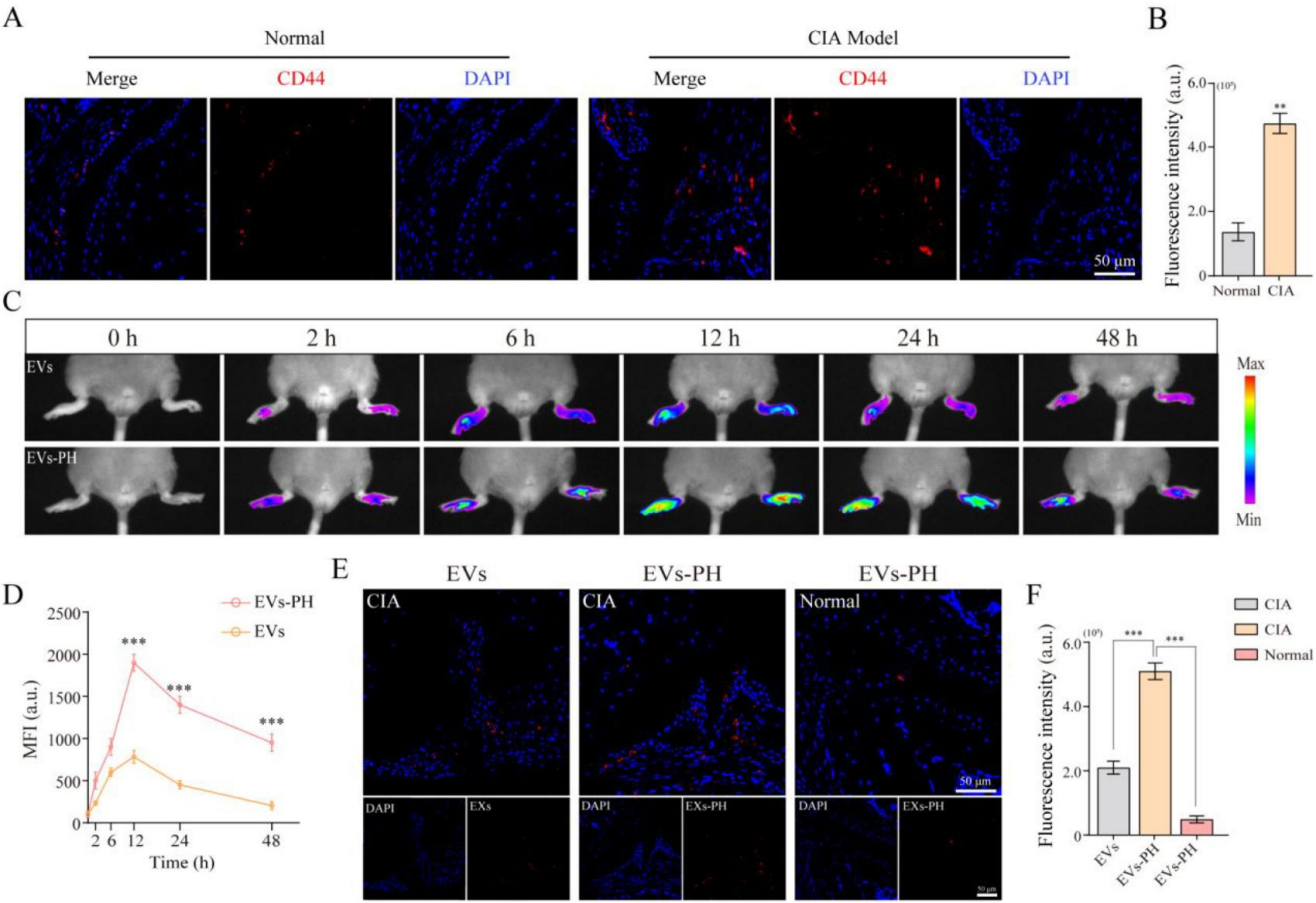
Active targeting capability of EVs–PH toward inflammatory sites in CIA mice. (**A**) Differential expression of CD44 in the joints of normal and CIA mice. (**B**) Quantitative analysis of CD44 fluorescence intensity in Figure 4A (*n* = 6). (**C**) The dynamic accumulation of EVs and EVs–PH at inflammatory sites was monitored by *in vivo* imaging. (**D**) Quantitative statistical analysis of Figure 4C (*n* = 6). (**E**) Immunofluorescence detection of EVs and EVs–PH localized to inflammatory sites. (**F**) Quantitative analysis of EVs and EVs–PH fluorescence intensity in Figure 4E (*n* = 6). Data are mean ± SEM. Statistical analysis was done using two-tailed unpaired *t*-tests. *P < 0.05, **P < 0.01, ***P < 0.001.

To evaluate the targeting capacity of EVs–PH to inflammatory joints, we first assessed CD44 expression in a CIA model. Immunofluorescence analyses revealed markedly elevated CD44 levels in the articular tissues of CIA mice compared to normal controls (Figure 4A and B), establishing a molecular basis for HA-mediated targeting. Subsequent *in vivo* imaging tracked the spatiotemporal distribution of EVs and EVs–PH following systemic administration. EVs–PH accumulated more rapidly and persistently at inflamed joint sites than unmodified EVs, a behavior attributed to the synergistic effect of HA-mediated active targeting and PEG-induced prolonged circulation (Figure 4C and D). Further immunofluorescence analysis of joint sections confirmed significantly stronger fluorescence signals from EVs–PH colocalized with inflammatory lesions, whereas only minimal signal was detected in joints of healthy control mice (Figure 4E and F). These results not only validate the inflammation-specific targeting ability of EVs–PH but also indicate that its therapeutic efficacy is contingent upon the inflammatory microenvironment. These findings demonstrate that EVs–PH exhibits enhanced joint-homing capability driven by CD44–HA interaction and stabilized by PEGylation, highlighting its potential as a targeted delivery system for the treatment of inflammatory arthritis.

### The enhanced anti-inflammatory and antioxidant efficacy of Cur@EVs–PH

To enhance the therapeutic efficacy of EVs–PH and leverage the natural drug-loading capacity of EVs, we developed Cur@EVs–PH (EE: 26.5% ± 1.8%, DL: 4.75% ± 0.2%, *n* = 3) by encapsulating curcumin within the HA-functionalized vesicles. To determine whether Cur@EVs–PH exerts a synergistic effect, five experimental groups were established: control (PBS), free curcumin (Cur), empty EVs–PH (EVs–PH), physical mixture of free curcumin and empty EVs–PH (Cur+EVs–PH), and curcumin-loaded EVs–PH (Cur@EVs-PH) (Cur: 4.75 μg/mL, EVs: 100 μg/mL). Their effects on oxidative stress, inflammatory cytokine secretion and macrophage polarization were systematically evaluated in LPS-stimulated macrophages. At the level of oxidative stress, ROS staining (Supplementary Figure S3A and B) revealed that treatment with either Cur or EVs–PH alone conferred moderate improvement. The physical mixture (Cur+EVs–PH) showed only a marginal improvement over EVs–PH alone. Notably, Cur@EVs–PH treatment led to a marked reduction in ROS levels, significantly outperforming the physical mixture, suggesting a synergistic advantage of the Cur@EVs–PH formulation. A similar trend was observed in the detection of the oxidative damage product MDA (Supplementary Figure S3C). Regarding antioxidant enzyme activity, quantitative analysis of SOD and GSH-Px (Supplementary Figure S3D and E) showed that Cur or EVs–PH alone exerted modest antioxidant effects, whereas Cur@EVs–PH significantly enhanced antioxidant enzyme activity, with efficacy notably superior to that of the Cur+EVs–PH group. In terms of inflammatory regulation, Cur@EVs–PH also demonstrated the most potent effects. Compared with the physical mixture, Cur@EVs–PH more effectively suppressed the expression of pro-inflammatory cytokines IL-1β, TNF-α and IL-6 (Supplementary Figure S3F), while more strongly promoting the anti-inflammatory cytokines IL-4 and IL-10 (Supplementary Figure S3G), further supporting its synergistic anti-inflammatory properties. Regarding macrophage polarization, Cur@EVs–PH treatment significantly downregulated the transcription of the M1 marker iNOS (Supplementary Figure S3H) and upregulated the transcription of the M2 marker Arg1 (Supplementary Figure S3I), with regulatory effects markedly exceeding those of the physical mixture and other individual treatments. Collectively, these results demonstrate that Cur@EVs–PH exhibits synergistic advantages over physical mixtures in alleviating oxidative stress, suppressing inflammation and modulating macrophage polarization. The therapeutic efficacy of Cur@EVs–PH is not merely additive but arises from the synergistic integration of targeted delivery by the carrier and the intrinsic bioactivity of curcumin, achieving a “1 + 1>2” synergistic effect.

To further elucidate the mechanism, we assessed Nrf2 activation in LPS‑induced macrophages. Compared to PBS treatment, Cur@EVs‑PH significantly promoted Nrf2 translocation into the nucleus (Supplementary Figure S4A and B), confirming its activation. This was accompanied by marked upregulation of HO‑1 at both transcriptional and translational levels, as determined by qPCR and Western blot (Supplementary Figure S4C–E). Additionally, Cur@EVs‑PH significantly increased the mRNA expression of *NQO1* and *GCLC* (Supplementary Figure S4F), two well‑characterized Nrf2 target genes. These data collectively demonstrate that Cur@EVs‑PH activates the Nrf2 signaling pathway, leading to enhanced expression of antioxidant genes.

### Cur@EVs–PH alleviates disease progression in CIA mice, demonstrating a therapeutic effect

**Figure 5:**
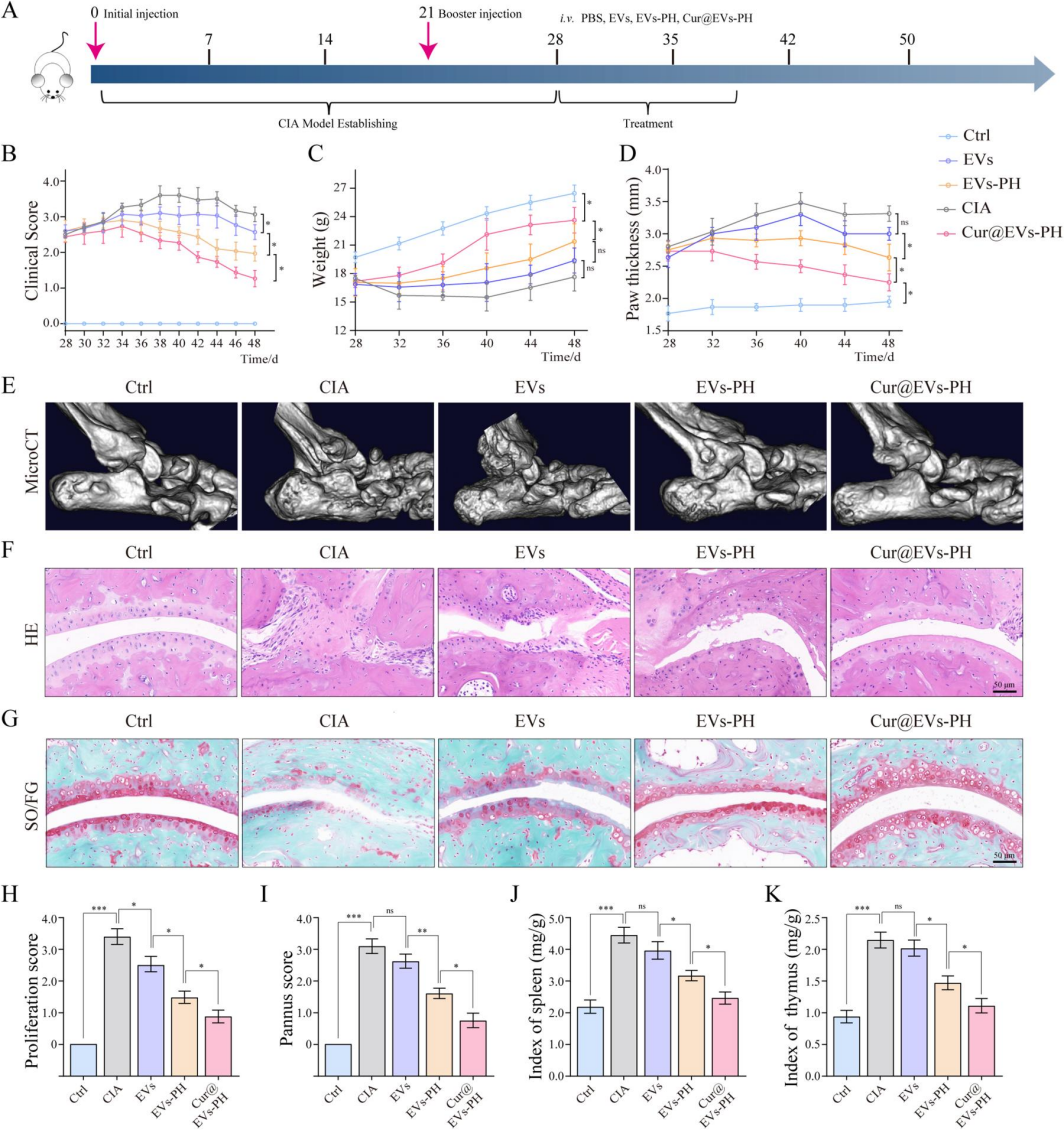
Cur@EVs–PH alleviates disease progression in CIA mice, demonstrating a therapeutic effect. (**A**) Timeline of CIA model establishment and treatment in mice. (**B**) Dynamic RA clinical scores in each group of mice (*n* = 6). (**C**) Body weight changes over time in each group of mice (*n* = 6). (**D**) Dynamic monitoring of hind limb swelling in each group of mice (*n* = 6). (**E**) Micro-CT three-dimensional reconstructions of ankle joints in each group. (**F**) H&E staining of joint inflammatory lesion sites in different mouse groups (*n* = 6). (**G**) SO/FG staining of joint inflammatory lesion sites in different mouse groups (*n* = 6). (**H**) Quantitative assessment of proliferation score based on pathological sections from different mouse groups (*n* = 6). (**I**) Quantitative assessment of pannus score based on pathological sections from different mouse groups (*n* = 6). (**J**) Quantification of spleen index in different mouse groups (*n* = 6). (**K**) Quantification of thymus index in different mouse groups (*n* = 6). Data are mean ± SEM. Statistical analysis was done using two-tailed unpaired *t*-tests. ns: not significant, *P < 0.05, **P < 0.01, ***P < 0.001.

We further evaluated the therapeutic potential of Cur@EVs–PH in a mouse model of CIA. Mice were randomly divided into five groups: normal, CIA+PBS, CIA+EVs, CIA+EVs–PH and CIA+Cur@EVs–PH. We subsequently evaluated the therapeutic potential of Cur@EVs–PH using a well-established CIA model, with the experimental timeline outlined in Figure 5A. Dynamic clinical assessment revealed that EVs–PH significantly improved RA clinical scores compared to the PBS control, while unmodified EVs showed only limited effects. Notably, Cur@EVs–PH treatment resulted in the most substantial and sustained clinical improvement throughout the observation period (Figure 5B). Body weight monitoring showed that all CIA model groups exhibited a significant decrease in body weight. Compared to the PBS group, EV treatment did not lead to statistically significant improvement. However, both EVs–PH and Cur@EVs–PH significantly ameliorated weight loss, with Cur@EVs–PH demonstrating the best therapeutic effect (Figure 5C). Longitudinal measurement of hind limb swelling demonstrated that EVs–PH effectively attenuated joint swelling, with Cur@EVs–PH exhibiting the most pronounced protective effect (Figure 5D), which was further corroborated by representative photographic evidence (Supplementary Figure S5A). Micro-CT images revealed that the Ctrl exhibited smooth articular surfaces and intact joint architecture with no evidence of bone erosion. In contrast, the CIA displayed typical RA-associated bone destruction, characterized by rough articular surfaces, severe bone erosion and notable osteophyte formation in some regions. Treatment with EVs or EVs–PH partially alleviated these pathological changes, showing limited improvement in joint structure. Notably, the Cur@EVs–PH group exhibited the most pronounced protective effect, with smoother articular surfaces and markedly reduced bone erosion, as clearly visualized in the reconstructed images (Figure 5E). These results demonstrate that Cur@EVs–PH effectively inhibits joint bone destruction in CIA mice, with superior efficacy compared to EVs or EVs–PH alone, further supporting the synergistic therapeutic advantage of this Cur-loaded exosome platform in mitigating RA-associated bone erosion. Histopathological evaluation provided compelling structural evidence for the therapeutic benefits. H&E staining revealed that EVs–PH substantially reduced inflammatory cell infiltration and synovial hyperplasia, while Cur@EVs–PH nearly restored normal joint architecture (Figure 5F). Correspondingly, SO/FG staining confirmed better preservation of cartilage and bone structure in the Cur@EVs–PH group (Figure 5G). Quantitative pathological assessment showed significantly lower proliferation scores (Figure 5H) and pannus formation (Figure 5I) in both EVs–PH and Cur@EVs–PH groups, with the latter achieving the most remarkable reduction. At the systemic level, both EVs–PH and Cur@EVs–PH normalized the elevated spleen and thymus indices commonly observed in CIA mice, while unmodified EVs showed no significant modulatory effect (Figure 5J and K), underscoring the critical importance of HA-mediated targeting in achieving systemic immunomodulation. In summary, Cur@EVs–PH integrates the advantages of targeted vesicle delivery and potent anti-inflammatory cargo, resulting in optimized therapeutic outcomes against inflammatory arthritis.

### Cur@EVs–PH ameliorates RA by alleviating inflammation and reducing oxidative stress

**Figure 6:**
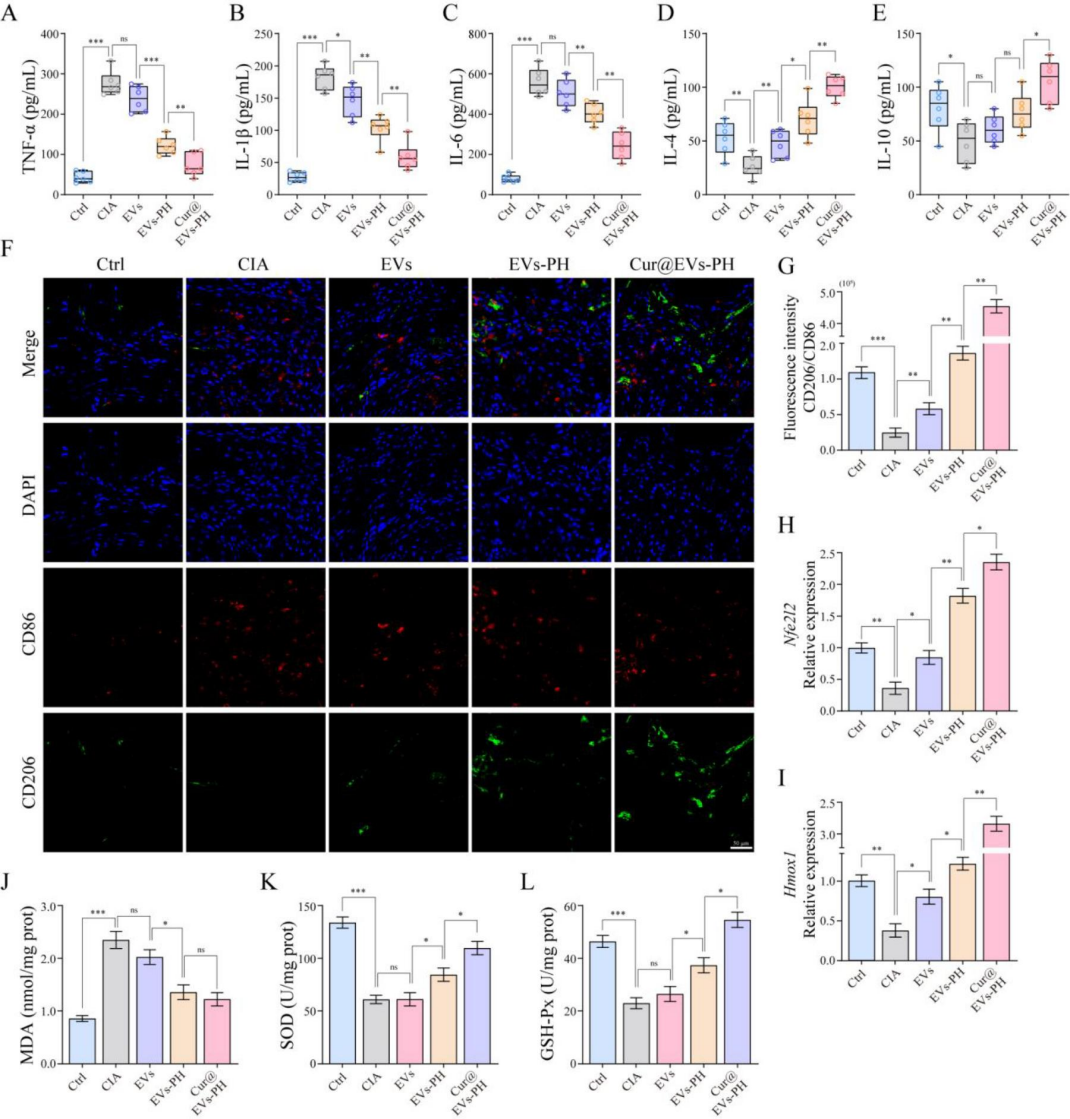
Cur@EVs–PH ameliorates RA by alleviating inflammation and reducing oxidative stress. (**A**–**C**) Comparative analysis of pro-inflammatory factors (IL-1β, TNF-α and IL-6) expression in each group of mice (*n* = 6). (**D** and **E**) comparative analysis of anti-inflammatory factors (IL-4 and IL-10) expression in each group of mice (*n* = 6). (**F**) Fluorescence imaging of macrophage polarization at arthritic sites in the joints of mice from each group. (**G**) Quantitative analysis of CD206/CD86 fluorescence intensity in Figure 6F (*n* = 6). (**H**) Differential expression of *Nfe2l2* at arthritic sites in the joints of mice from each group (*n* = 6). (**I**) Differential expression of *Hmox1* at arthritic sites in the joints of mice from each group (*n* = 6). (**J**) Quantification of MDA at arthritic sites in the joints of mice from each group (*n* = 6). (**K**) Quantification of SOD at arthritic sites in the joints of mice from each group (*n* = 6). (**L**) Quantification of GSH-Px at arthritic sites in the joints of mice from each group (*n* = 6). Data are mean ± SEM. Statistical analysis was done using two-tailed unpaired *t*-tests. ns: not significant, *P < 0.05, **P < 0.01, ***P < 0.001.

To investigate the underlying mechanisms by which Cur@EVs–PH ameliorates RA, we systematically evaluated its anti-inflammatory, immunomodulatory and antioxidant effects. As shown in Figure 6A–C, the expression of pro-inflammatory cytokines (IL-1β, TNF-α and IL-6) was significantly elevated in the joints of CIA model mice. EVs treatment only slightly reduced IL-1β and TNF-α levels and had no significant effect on IL-6. In contrast, EVs–PH markedly suppressed all three cytokines, while Cur@EVs–PH exhibited the strongest anti-inflammatory efficacy, restoring IL-1β, TNF-α and IL-6 levels close to those of the normal group. Conversely, the expression of anti-inflammatory cytokines (IL-4 and IL-10) was most prominently enhanced in the Cur@EVs–PH group (Figure 6D and E), indicating its ability to effectively rebalance the inflammatory microenvironment. Further macrophage polarization assays (Figure 6F–G) revealed that Cur@EVs–PH significantly increased fluorescence intensity of the M2 marker CD206 while suppressing the M1 marker CD86, demonstrating its role in promoting a shift toward the anti-inflammatory macrophage phenotype, thereby alleviating joint inflammation. Regarding antioxidant mechanisms, Cur@EVs–PH notably upregulated the expression of Nrf2 (encoded by *Nfe2l2*) and its downstream gene *Hmox1* (Figure 6H and I), activating a key antioxidant signaling pathway. Meanwhile, the oxidative stress marker MDA was significantly reduced (Figure 6J), while the activities of antioxidant enzymes SOD and GSH-Px were markedly elevated (Figure 6K and L) in the Cur@EVs–PH group, indicating substantial mitigation of oxidative damage in joint tissues. In summary, our findings elucidate the multi-mechanistic therapeutic effects of Cur@EVs–PH in RA through modulation of inflammatory responses, immune phenotype switching and enhancement of antioxidant defense.

### Safety evaluation of Cur@EVs–PH

To evaluate the biosafety of the different formulations, key biochemical markers related to liver and kidney function as well as cellular integrity—including alkaline phosphatase (ALP), aspartate aminotransferase (AST), alanine aminotransferase (ALT), blood urea nitrogen (BUN) and lactate dehydrogenase (LDH)—were measured across treatment groups. The results showed no significant differences in the levels of ALP, AST, ALT, BUN, or LDH among the EVs, EVs–PH and Cur@EVs–PH groups (Supplementary Figure S6A–E). These findings indicate that neither the modification of EVs with PH nor the subsequent loading of Cur introduced detectable toxic effects on hepatic or renal function, confirming the good biocompatibility and preliminary safety of all engineered vesicle systems.

## Discussion

Our study developed a Cur@EVs–PH composite system based on ginseng stems and leaves-derived EVs through HA modification and curcumin (Cur) loading, which exhibits inflammatory targeting, antioxidant and immunomodulatory functions. Systematic experiments validated its significant therapeutic efficacy in RA and elucidated its multi-mechanistic synergistic mode of action.

First, we demonstrated that functional PH modification enhanced the targeting capability of EVs. Both *in vivo* imaging and histological section analysis showed that EVs–PH exhibited stronger accumulation and longer retention time in inflamed joints. This characteristic can be attributed to the specific binding between HA and the highly expressed CD44 receptors at inflammatory sites [45], as well as the prolonged circulation effect conferred by PEGylation [46]. Notably, when the CD44 blocker IM7 was applied, the therapeutic effect of EVs–PH was significantly diminished, directly proving the crucial role of CD44-HA interaction in this targeting system. This active targeting capability not only increases drug concentration at the lesion sites but also lays the foundation for reducing systemic side effects.

CD44 is a widely expressed transmembrane glycoprotein that primarily serves as the receptor for HA. It is involved in diverse physiological and pathological processes, including cell–cell and cell–matrix adhesion, cell migration, proliferation and signal transduction [47]. During the progression of RA, the synovium is rich in pro-inflammatory cytokines. These cytokines not only recruit macrophages into the synovial tissue but also potently activate key intracellular signaling pathways in macrophages, such as NF-κB and MAPK. The activation of these pathways directly initiates and upregulates the transcription of the CD44 gene, leading to a marked increase in CD44 expression [48]. On the other hand, elevated CD44 enhances the adhesion and migration of immune cells, promoting their retention at the inflammatory site and establishing a positive feedback loop. From a materials design perspective, this mechanism provides a valuable opportunity for targeted drug delivery, creating an ideal biological foundation for drug delivery systems that utilize HA as a targeting ligand.

At the mechanistic level, Cur@EVs–PH demonstrated multi-pathway synergistic therapeutic advantages. In terms of inflammatory regulation, the system significantly suppressed the expression of pro-inflammatory cytokines such as IL-1β, TNF-α and IL-6, while promoting the secretion of anti-inflammatory factors including IL-4 and IL-10. More importantly, we observed that Cur@EVs–PH effectively promoted macrophage polarization from the pro-inflammatory M1 phenotype to the anti-inflammatory M2 phenotype, an effect visually confirmed by fluorescence staining of joint tissues. This switch in immune phenotype is of great significance for breaking the vicious cycle of inflammation in RA. Importantly, we propose that this amelioration of the oxidative microenvironment is instrumental in facilitating the observed repolarization of macrophages from the M1 to the M2 phenotype. The shift toward the M2 phenotype, visually confirmed in tissue, further contributes to resolving inflammation and may, in turn, reduce the generation of ROS, creating a positive feedback loop that breaks the vicious cycle of RA progression. Therefore, the therapeutic efficacy of Cur@EVs–PH likely stems from its dual capacity to concurrently target oxidative stress and inflammation, where these two mechanisms are interdependent and mutually reinforcing.

Regarding antioxidant mechanisms, Cur@EVs–PH significantly activated the Nrf2/HO-1 signaling pathway. As a core regulatory pathway of the cellular antioxidant defense system, Nrf2 activation directly led to enhanced activities of antioxidant enzymes such as SOD and GSH-Px, along with reduced MDA levels [49]. It is worth noting that while EVs–PH alone exhibited certain antioxidant effects, Cur@EVs–PH showed more pronounced efficacy, indicating a favorable synergistic effect between curcumin loading and the inherent active components of EVs. This antioxidant action not only alleviates direct oxidative damage to joint tissues but may also indirectly influence the activation of inflammatory signaling pathways by regulating redox balance [50].

Compared with previous studies, the innovation of this research lies in the organic integration of the biological activity of natural source EVs, the precision of targeting modification, and the synergy of efficient drug loading [51]. Conventional EVs therapies often face challenges of insufficient targeting and limited efficacy, while nanodrug delivery systems lack the biological functions and good biocompatibility of natural EVs [52, 53]. The strategy adopted in this study not only overcomes these limitations but also fully utilizes the inherent antioxidant properties of ginseng stems and leaves-derived EVs and the potent anti-inflammatory activity of Cur, achieving exceptional combined therapeutic efficacy.

The disease-tailored nanoplatform based on plant-derived EVs is designed by targeting the specific pathological changes at lesion sites. Leveraging its CD44-targeting design, Cur@EVs–PH holds potential for application in multiple diseases. For instance, in inflammatory bowel disease, the platform can deliver therapeutics efficiently to intestinal inflammatory sites by targeting CD44-high immune cells such as macrophages and activated T cells; in psoriasis, it can target activated keratinocytes and infiltrating immune cells [54, 55]. Moreover, it shows promise for osteoarthritis, other autoimmune disorders and the tumor immune microenvironment by directing therapy to CD44 overexpressing cells [56, 57]. With favorable biocompatibility, drug-loading flexibility and a modular nature, the platform can be rapidly adapted to different therapeutic needs by exchanging cargoes (e.g. nucleic acid drugs, small-molecule inhibitors), offering a versatile technological platform for next-generation targeted immunomodulatory therapies.

However, this study still has some limitations. First, although we confirmed the short-term efficacy and safety of Cur@EVs–PH, its long-term toxicity and immunogenicity require further evaluation. Second, the large-scale production and quality control of EVs remain critical bottlenecks hindering clinical translation. Additionally, the universality of EVs–PH application in different inflammatory models and its synergistic effects with other antirheumatic drugs warrant further investigation.

## Conclusion

This study successfully developed a multifunctional EV delivery system with integrated inflammatory targeting, antioxidant and immunomodulatory capabilities. Through systematic *in vitro* and *in vivo* experiments, we demonstrated the significant therapeutic efficacy of Cur@EVs–PH in RA and elucidated its molecular mechanisms of action, including regulation of inflammatory responses, promotion of macrophage M2 polarization and activation of the Nrf2/HO-1 pathway. These findings not only provide new ideas and methods for the treatment of RA but also offer a theoretical basis and practical references for the application of EVs-based targeted therapy platforms in inflammatory diseases.

## Supplementary Material

rbag078_Supplementary_Data

## Data Availability

The data that support the findings of this study are available from the corresponding author upon reasonable request.
