# Supplementary material for: M1 macrophage-targeted engineered ginseng stems and leaves-derived extracellular vesicles delivery system for alleviating rheumatoid arthritis
Source: Regen Biomater. 2026 Apr 26;13:rbag078. doi: 10.1093/rb/rbag078 (PMC13213593; doi:10.1093/rb/rbag078)
Supplement: rbag078_Supplementary_Data [file rbag078_supplementary_data.docx]

***Supplemental Material***

**M1 Macrophage-Targeted Engineered Ginseng Stems and Leaves-Derived Extracellular Vesicles Delivery System for Alleviating Rheumatoid Arthritis**

Chuanjie Zhang^1^^†^, Yingjie Wang^2†^, Xiaoyu Jiang^3†^, Dake Wang^1^, Yajiang Yuan^4^, Jianye Li^1^, Weiran Gao^4*^, Housen Jiang^5*^, Xifan Mei^1*^

^1^ The Third Affiliated Hospital of Jinzhou Medical University, Jinzhou 121000, China.

^2^ Department of Ophthalmology, Beijing Chaoyang Hospital Affiliated to Capital Medical University, Beijing, 100020, China.

^3^ Shandong University of Traditional Chinese Medicine, Jinan 250014, China

^4^ The First Hospital of Jinzhou Medical University, Jinzhou, 121001, China.

^5^ Department of Hand Foot Orthopedics, First Affiliated Hospital of Shandong Second Medical University, Weifang, 261000, China.

* Corresponding authors:

Xifan Mei, E-mail: meixifan1971@163.com

Housen Jiang, E-mail: jhs115612@sina.com

Weiran Gao, E-mail: smilegao_520@163.com

^†^ Chuanjie Zhang, Yingjie Wang and Xiaoyu Jiang contributed equally to this work.

**
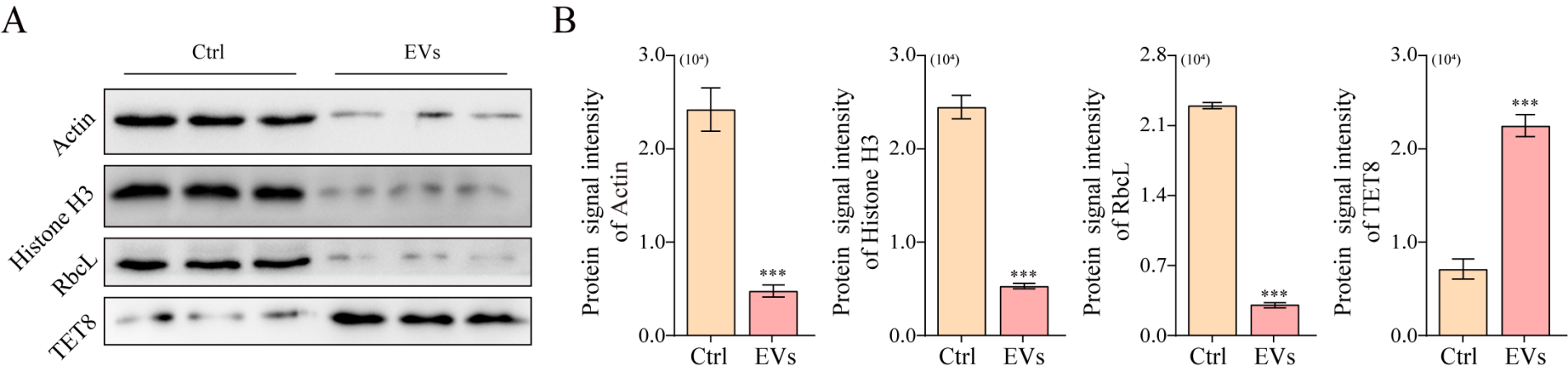
**

**Figure. S1. Purity validation of EVs by Western blotting.** (A) Differential expression of Actin, Histone H3, RbcL and TET8 between the ginseng stem and leaf total lysate (Ctrl) and EVs as determined Western Blot. (B) Quantitative Western blot analysis of Actin, Histone H3, RbcL and TET8 in Figure R1A (n=3).

**
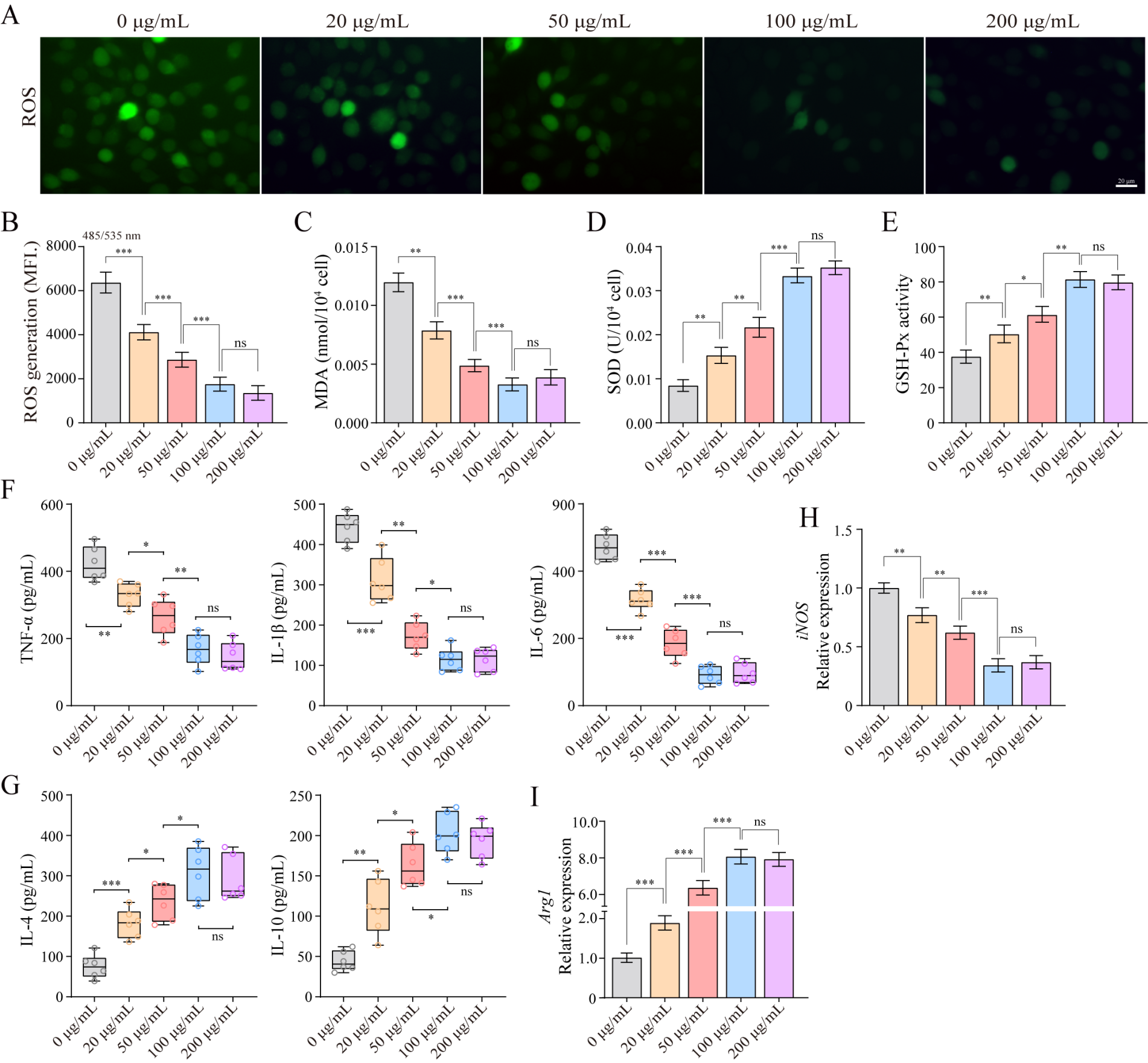
**

**Figure. R2. EVs-PH exerts dose-dependent effects on both inflammatory response and oxidative stress.** (A) Immunofluorescence analysis of ROS in M1 macrophages across various groups. (B) Quantitative analysis of ROS fluorescence intensity in Figure 3A (n=6). (C) Quantification of MDA in M1 macrophages across various groups (n=6). (D) Quantification of SOD in M1 macrophages across various groups (n=6). (E) Quantification of GSH-Px in M1 macrophages across various groups (n=6). (F) Comparative analysis of pro-inflammatory factors (IL1-β, TNF-α and IL-6) expression in M1 macrophages from various groups (n=6). (G) Comparative analysis of anti-inflammatory factors (IL4 and IL-10) expression in M1 macrophages from various groups (n=6). (H) Differential expression of *iNOS* in M1 macrophages among different groups (n=6). (I) Differential expression of *Arg1* in M1 macrophages among different groups (n=6). Data are mean ± SEM. Statistical analysis was done using two-tailed unpaired t-tests. ns: not significant, * P < 0.05, ** P < 0.01, *** P < 0.001.

**
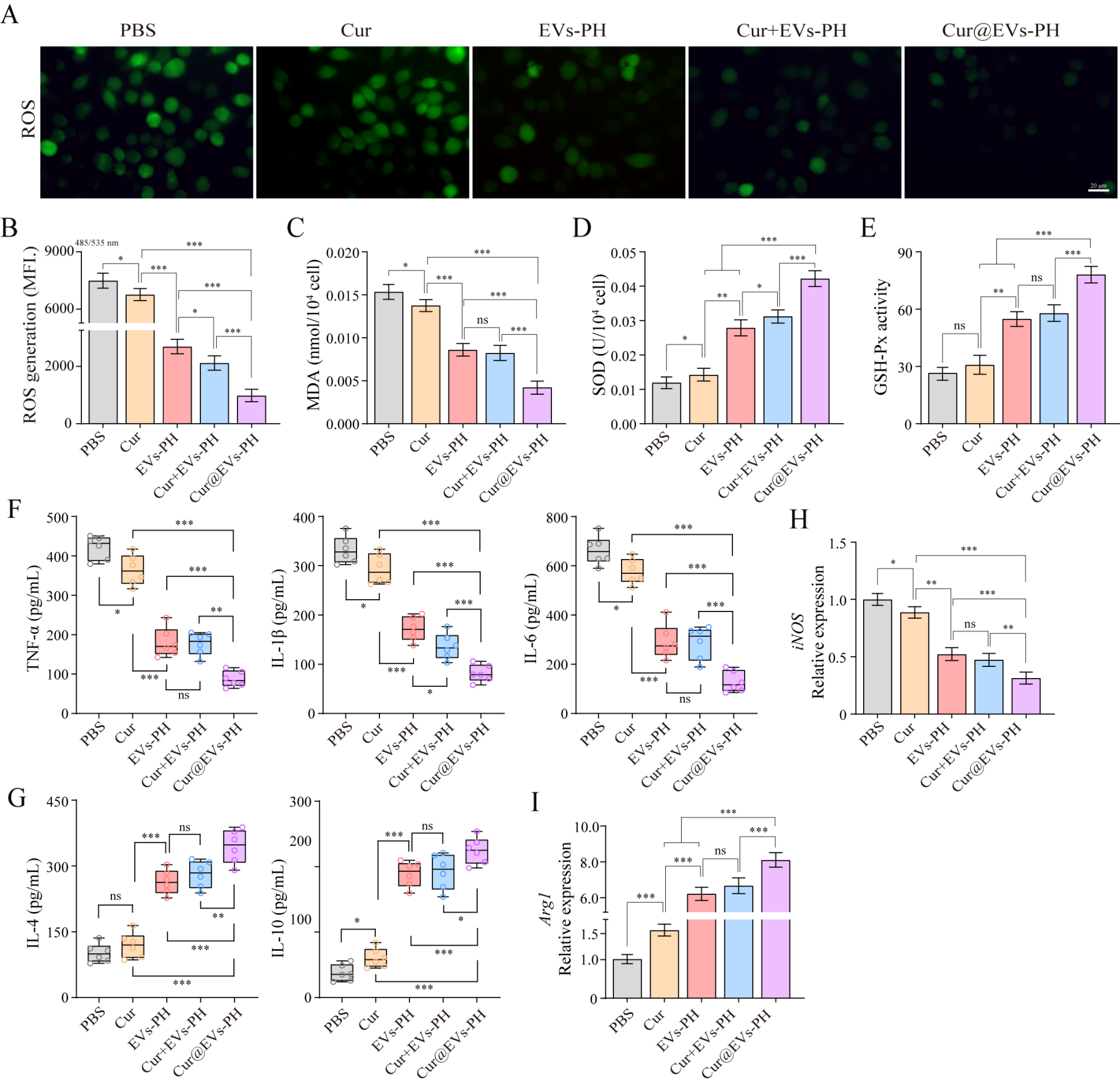
**

**Figure. S3. The enhanced anti-inflammatory and antioxidant efficacy of Cur@EVs-PH.** (A) Immunofluorescence analysis of ROS in M1 macrophages across various groups. (B) Quantitative analysis of ROS fluorescence intensity in Figure 3A (n=6). (C) Quantification of MDA in M1 macrophages across various groups (n=6). (D) Quantification of SOD in M1 macrophages across various groups (n=6). (E) Quantification of GSH-Px in M1 macrophages across various groups (n=6). (F) Comparative analysis of pro-inflammatory factors (IL1-β, TNF-α and IL-6) expression in M1 macrophages from various groups (n=6). (G) Comparative analysis of anti-inflammatory factors (IL4 and IL-10) expression in M1 macrophages from various groups (n=6). (H) Differential expression of iNOS in M1 macrophages among different groups (n=6). (I) Differential expression of Arg1 in M1 macrophages among different groups (n=6). Data are mean ± SEM. Statistical analysis was done using two-tailed unpaired t-tests. ns: not significant, * P < 0.05, ** P < 0.01, *** P < 0.001.

**
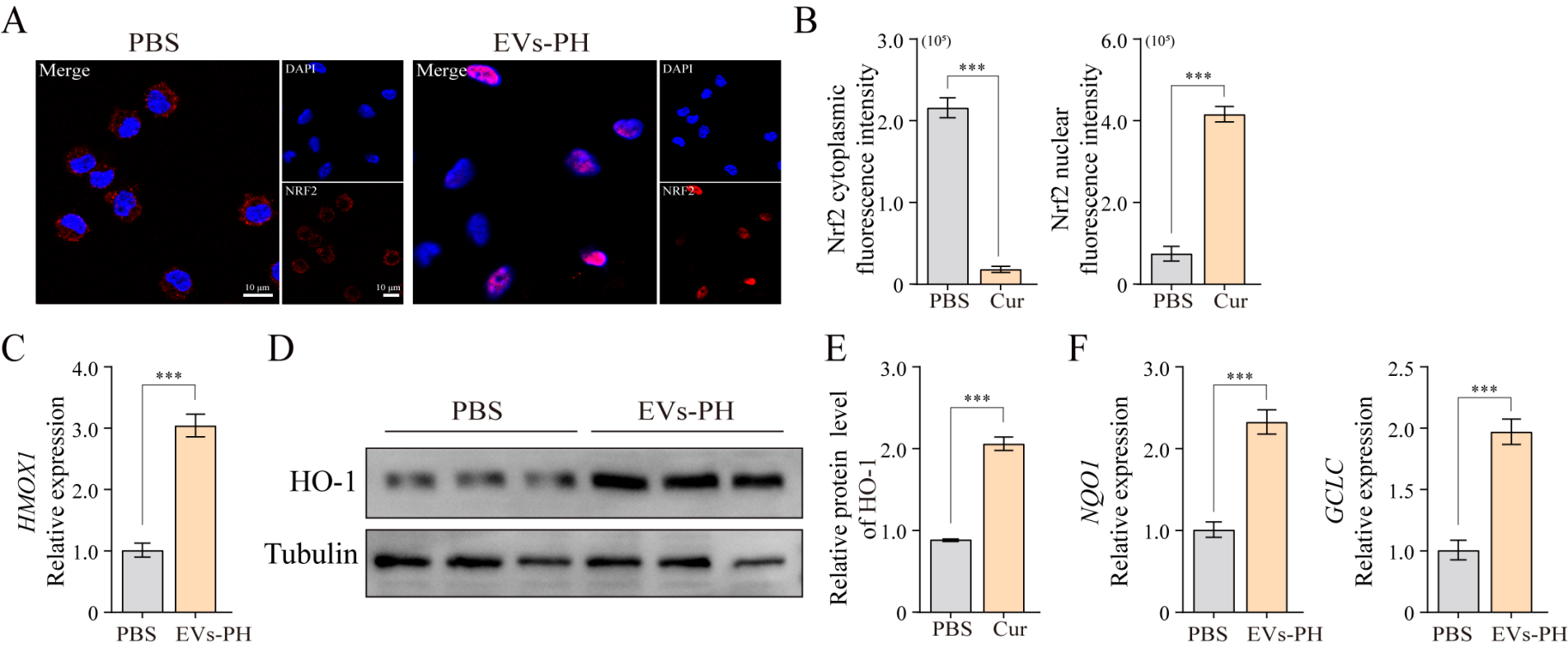
**

**Figure. S4. Cur@EVs-PH activates the Nrf2/HO-1 signaling pathway in macrophages.** (A) Immunofluorescence analysis of Nrf2 in LPS-induced macrophages across various groups. (B) Statistical analysis of nuclear and cytoplasmic Nrf2 in Figure R6A (n=6). (C) Differential expression of HMOX1 in LPS-induced macrophages among different groups (n=6). (D) Differential expression of HO-1 in LPS-induced macrophages among different groups. (E) Quantitative Western blot analysis of HO-1 in Figure 2A (n=3). (F) Differential expression of *NQO1* and *GCLC* in LPS-induced macrophages among different groups (n=6).

**
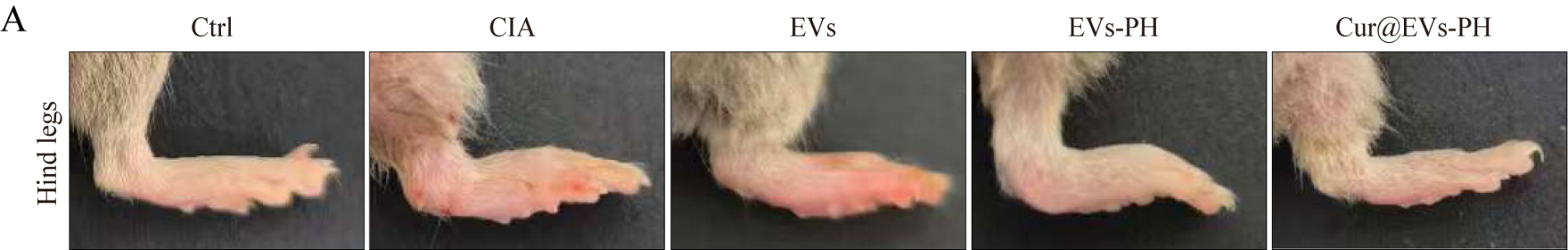
**

**Figure. S5. Cur@EVs-PH alleviates disease progression in CIA mice, demonstrating a therapeutic effect.** (A) Representative images showing the severity of hind limb swelling in each group of mice (n=6).

**
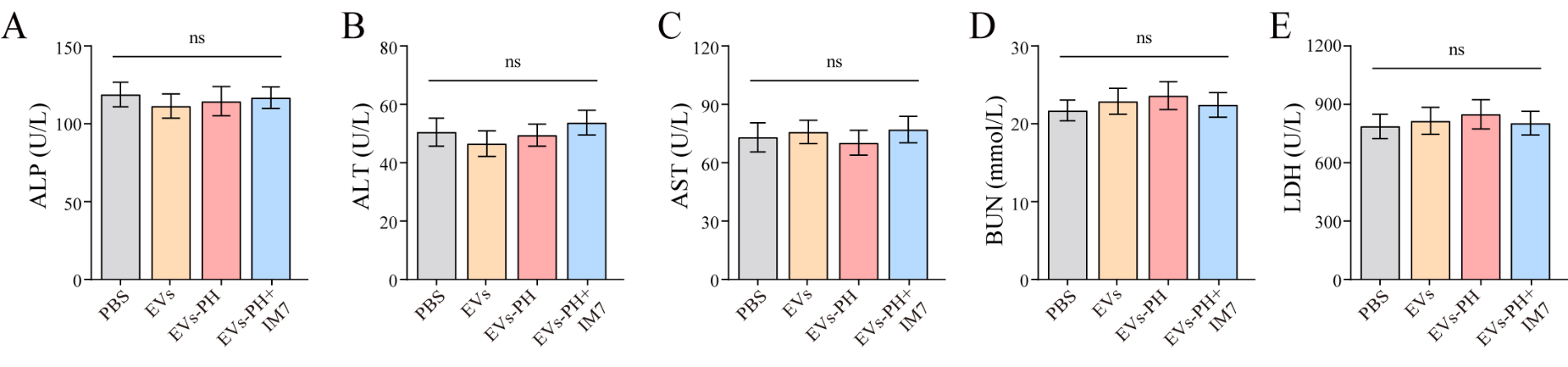
**

**Figure. S6. Biocompatibility assessment of Cur@EVs-PH, EVs-PH, and EVs.**

(A-E) Assessment of a panel of serum safety markers (ALP: Alkaline Phosphatase, AST: Aspartate Aminotransferase, ALT: Alanine Aminotransferase, BUN: Blood Urea Nitrogen, LDH: Lactate Dehydrogenase) (n=6). Data are mean ± SEM. Statistical analysis was done using two-tailed unpaired t-tests. ns: not significant.

**Table S1. Standard of pathological score for ankle joint.**

| Score | 0 | 1 | 2 | 3 |
| --- | --- | --- | --- | --- |
| cell proliferation | none | slight proliferation  in 2~4 layers | moderate cell proliferation  in more than 4 layers | excessive cell proliferation  cartilage erosion  joint space disappears |
| panus | none | in 2 places | 4 sites  cartilage erosion | more than 4 parts  or in large areas in 2 parts |
| cell erosion | none | 1~2 cell erosion foci | 2-5 local cell erosion foci | more than 5 cells erosion foci  spread to the joint capsule |
| inflammation | normal | Mild inflammation  1 aggregate or a few scattered inflammatory cell infiltration | moderate inflammation  2 or more leukocyte aggregates | severe inflammation  plenty of cells infiltrate  plenty of WBCs gather obviously scattered infiltration |

**Table S2. Primers required for qPCR.**

| Primer name | From 5’ to 3’ |
| --- | --- |
| GAPDH mouse F | ACGGATTTGGTCGTATTGGG |
| GAPDH mouse R | GTCGGAGATTCGTAGCTGGA |
| Cd36 mouse F | ATGGGCTGTGATCGGAACTG |
| Cd36 mouse R | GTCTTCCCAATAAGCATGTCTCC |
| Timd4 mouse F | CCGGTGACTTTGCCTTGTCAT |
| Timd4 mouse R | CTCTGCATTGCACTTGGAATTG |
| Msr1 mouse F | GCACAATCTGTGATGATCGCT |
| Msr1 mouse R | CCCAGCATCTTCTGAATGTGAA |
| Mertk mouse F | CAGGGCCTTTACCAGGGAGA |
| Mertk mouse R | TGTGTGCTGGATGTGATCTTC |
| Stab2 mouse F | AGCTGCTGCCTTTAATCCTCA |
| Stab2 mouse R | ACTCCGTCTTGATGGTTAGAGTA |
| Nfe2l2 mouse F | CTGAACTCCTGGACGGGACTA |
| Nfe2l2 mouse R | CGGTGGGTCTCCGTAAATGG |
| Hmox1 mouse F | AAGCCGAGAATGCTGAGTTCA |
| Hmox1 mouse R | GCCGTGTAGATATGGTACAAGGA |
